# Automatic Detection of Abnormal EEG Signals Using WaveNet and LSTM

**DOI:** 10.3390/s23135960

**Published:** 2023-06-27

**Authors:** Hezam Albaqami, Ghulam Mubashar Hassan, Amitava Datta

**Affiliations:** 1 Department of Computer Science and Software Engineering, The University of Western Australia, Perth 6009, Australia; ghulam.hassan@uwa.edu.au (G.M.H.); amitava.datta@uwa.edu.au (A.D.); 2 Department of Computer Science and Artificial Intelligence, University of Jeddah, Jeddah 201589, Saudi Arabia

**Keywords:** electroencephalogram (EEG), deep learning, CNN, LSTM, WaveNet

## Abstract

Neurological disorders have an extreme impact on global health, affecting an estimated one billion individuals worldwide. According to the World Health Organization (WHO), these neurological disorders contribute to approximately six million deaths annually, representing a significant burden. Early and accurate identification of brain pathological features in electroencephalogram (EEG) recordings is crucial for the diagnosis and management of these disorders. However, manual evaluation of EEG recordings is not only time-consuming but also requires specialized skills. This problem is exacerbated by the scarcity of trained neurologists in the healthcare sector, especially in low- and middle-income countries. These factors emphasize the necessity for automated diagnostic processes. With the advancement of machine learning algorithms, there is a great interest in automating the process of early diagnoses using EEGs. Therefore, this paper presents a novel deep learning model consisting of two distinct paths, WaveNet–Long Short-Term Memory (LSTM) and LSTM, for the automatic detection of abnormal raw EEG data. Through multiple ablation experiments, we demonstrated the effectiveness and importance of all parts of our proposed model. The performance of our proposed model was evaluated using TUH abnormal EEG Corpus V.2.0.0. (TUAB) and achieved a high classification accuracy of 88.76%, which is higher than in the existing state-of-the-art research studies. Moreover, we demonstrated the generalization of our proposed model by evaluating it on another independent dataset, TUEP, without any hyperparameter tuning or adjustment. The obtained accuracy was 97.45% for the classification between normal and abnormal EEG recordings, confirming the robustness of our proposed model.

## 1. Introduction

### 1.1. Background

The electrical activity within the brain provides critical insights into its functionality and overall human well-being. Electroencephalogram (EEG) serves as a diagnostic tool for numerous neurological disorders [1]. As a noninvasive instrument, EEG signals facilitate the examination of diverse brain disorders and foster a deeper comprehension of the human brain. However, no single mathematical or biological model can fully elucidate the array of EEG patterns, rendering the understanding of EEG signals primarily a phenomenological medical domain [2]. Conventionally, trained professionals such as neurologists and physicians are solely responsible for visually analyzing lengthy EEG records and identifying normal or abnormal activities [2]. The immense volume of EEG recordings has placed a significant burden on clinicians and researchers [2]. Consequently, there is an urgent need to develop automated systems to assist in diagnostic evaluation and reduce their workload.

In response to this demand, recent advancements in machine learning techniques and computing power have spurred a growing interest in leveraging these technologies to interpret EEG data automatically. Indeed, researchers have made significant progress in applying these methods to detect and classify a broad spectrum of neurological disorders, including epileptic seizure prediction, detection [3,4], and classification [5]; Alzheimer’s Disease (AD) detection [6]; sleep disorder detection [7,8]; Attention Deficit Hyperactivity Disorder (ADHD) detection [9,10]; and general abnormality detection using EEGs [11]. A diverse array of machine learning methods has been utilized to address the challenges inherent in interpreting EEG data for various disorders [12]. These include deep learning approaches such as Convolutional Neural Networks (CNNs) [13] and Recurrent Neural Networks (RNNs) [11], alongside traditional techniques such as Support Vector Machines (SVMs) [14,15], K-nearest Neighbor (KNN) [10], Random Forests (RF) [16,17], Linear Discriminant Analysis (LDA) [18,19,20], Gradient Boosting Decision Tree (GBDT) [16,21], and Logistic Regression (LG) [22].

Building on these advancements in machine learning, researchers have explored various methods of feature extraction to process raw EEG signals. Techniques such as Wavelet Packet Decomposition (WPD) [21,23], Discrete Wavelet Transform (DWT) [16,24,25], Dual-Tree Complex Wavelet Transform (DTCWT) [26], Empirical Wavelet Transform (EWT) [15], Empirical Mode Decomposition (EMD) [15], Fast Fourier Transform (FFT) [27], Short-time Fourier Transform (STFT) [14,17,28], and entropy-based measures [25,29] have been employed. However, these methods necessitate specialized knowledge and extensive feature extraction and signal processing computation [12]. Furthermore, the process of feature extraction is both time-consuming and labor-intensive, demanding significant domain-specific expertise in each type of features [12,30].

Drawing inspiration from the success of deep learning methods in other fields, such as computer vision [31], Natural Language Processing (NLP) [32], and speech recognition [33], the focus of automatic EEG analysis has shifted from a feature-based domain to a deep feature learning domain [12]. This paradigm shift allows deep learning models to learn directly from raw EEG data, bypassing the need for feature extraction [30,34]. Such an approach holds the promise of substantially reducing expert workloads while maintaining the accuracy of EEG analysis.

In this study, we introduce a novel deep-learning architecture that combines a customized WaveNet–Long Short-Term Memory (LSTM) sub-model with LSTM components through serial concatenation, aiming to achieve highly accurate EEG anomaly detection. Previously employed in speech generation and reconstruction [35], the WaveNet model demonstrates a remarkable ability to learn discriminative features from deep convolution layers, efficiently utilizing multi-resolution dilated convolutions and various receptive fields.

### 1.2. Related Work

The development of automated clinical EEG analysis through the application of machine learning techniques represents an expanding area of research within the EEG domain [36]. For binary EEG pathology detection, identifying if the brain activity is normal or abnormal, most studies focused on Temple University Hospital (TUH) Abnormal EEG Corpus (TUAB) [37]. This dataset contains around 3000 EEG recordings, which have been carefully labeled by neurologists and students, making it a valuable resource [37].

Previous work for pathology detection can be broadly classified into two main categories: feature-based and end-to-end approaches [12]. The feature-based approach has traditionally relied on experts to extract relevant features from the raw data manually. Currently, there are numerous feature-based studies in the literature that discuss the automated detection of pathologies in EEG. Various studies have implemented methods based on time-frequency domain. For instance, WPD was employed in [21,23,38], while DWT was utilized in [12,16,24]. Other methods include CWT, as demonstrated in [12], and STFT, as used in [14]. In the frequency-domain, FFT-based feature extraction was employed in [27]. Finally, in [12,39], time-domain based features were utilized. Table 1 summarizes the feature-based approaches for EEG abnormality detection found in the literature. While previous studies have yielded successful results in the automated detection of pathologies in EEG, they remain labor-intensive and require specialty in each domain of features [12].

Conversely, the end-to-end approaches have demonstrated immense potential in expeditiously learning directly from raw data for the purpose of automated pathology detection. Among the various end-to-end deep learning models are CNNs, or so-called ConvNets [13], and RNNs [11]. Researchers have proposed several models for detecting abnormalities in EEG data, with varying accuracies. Schirrmeister et al. [13] proposed deep and shallow CNN models for detecting abnormalities in EEG data, achieving accuracies of 85.4% and 84.5%, respectively. Yildirim et al. [43] designed a 23-layer deep 1D-CNN model that attained 79.34% accuracy for the same task. Roy et al. [44] developed a 1D-CNN model consisting of stacked 1D-CNN layers followed by several Gated Recurrent Unit (GRU) layers, which were trained on EEG data for abnormality detection, enabling them to achieve an accuracy of 82.27%. Roy et al. [11], in another study, proposed a deep-gated RNN model, ChronoNet, which combined multiple 1D-CNNs with GRUs, achieving an accuracy of 86.57%. Gemein et al. [12] employed a Temporal Convolutional Network (TCN) model, suitable for sequence modeling and time-series analysis, and achieved an accuracy of 86.1%. Amin et al. [45] implemented the well-known CNN model, AlexNet, combined with SVM for identifying abnormalities in EEGs, reporting an accuracy of 87.32%. Khan et al. [46] proposed a hybrid model that integrated a CNN with an LSTM sub-model, achieving an accuracy of 85%. Recently, Kiessner et al. [47] trained different CNN-based models [12,13] on an extended version of the TUH dataset and tested them on a commonly used evaluation dataset [37], attaining an accuracy of 86.59%.

Table 2 shows an overview of related studies using end-to-end approaches. It can be observed that there are only a few studies directed at the problem of abnormal EEG detection, which indicates a strong need for more focused research to address this problem. Despite the promising results achieved by previous studies, there are still areas where improvements can be made. Previous state-of-the-art deep learning research studies used large models with high parameter counts, such as VGG19 [17] (143 million) and AlexNet [45] (61 million) [14]. Furthermore, most previous studies have used up to 10 min of EEG data, while neurologists typically make a determination by examining just the first few minutes of a recording [37]. As a result, in this study, we concentrate on analyzing the initial 60 s of an EEG, only to mimic real-world neurologists’ behavior. Moreover, most previous studies have only tested their proposed models on a single dataset, while we evaluated our proposed technique on multiple datasets.

### 1.3. Novelties and Contributions

Considering these limitations, we propose a WaveNet–LSTM architecture specifically designed for the binary classification of EEG data. Our approach seeks to address the above-mentioned issues by offering the following contributions:

- We propose a novel deep learning network that can effectively discriminate between normal and abnormal EEGs;
- Our proposed network incorporates WaveNet and LSTM to learn salient features from raw EEGs, without the need to manually extract features;
- Our model’s scalable structure effectively reduces both computational complexity and training duration, which is a significant advancement over traditional methods;
- The WaveNet architecture utilized in our study extracts high-level spatial features from the raw EEG data, while LSTM refines the context correlation of those features in their temporal pattern;
- Our proposed model was evaluated on the most extensive abnormal EEG database, TUAB (2.0.0); furthermore, the generalizability was evaluated across different databases;
- Our proposed solution utilizes a novel method incorporated in our study which generates additional data through the use of Time Reverse EEG data augmentation;
- We rigorously tested each component of the proposed model to identify their contribution to the final classification results;
- The proposed network achieved the best results in classifying normal and abnormal EEGs in a patient-independent scenario, utilizing only the initial duration of the signals. This can be attributed to the importance of integrating both the WaveNet and LSTM components for the feature learning process;
- To the best of our knowledge, this is the first study that employs WaveNet and LSTM in an innovative structure for the problem of EEG-based abnormality detection.

The structure of this article is as follows. Section 2 explains the datasets used, preprocessing steps, and a detailed introduction to our proposed model and performance evaluation metrics. In Section 3, we discuss our results and compare them with findings from other studies. Lastly, Section 4 concludes this paper with recommendations for future work.

## 2. Materials and Methods

In this study, we propose to use a hybrid WaveNet–LSTM-based model for the problem of automatic detection of EEG signals. We based our study on the TUAB EEG dataset [37]. In addition, we demonstrated the generalizability of our proposed model by rigorously testing our model across different patients. This section details the specifics of our research, covering the data we used, the preprocessing steps performed, the structure of the proposed deep learning architecture, and the ablation studies carried out to assess the performance of each component of our proposed model.

### 2.1. Datasets

**Figure 1:**
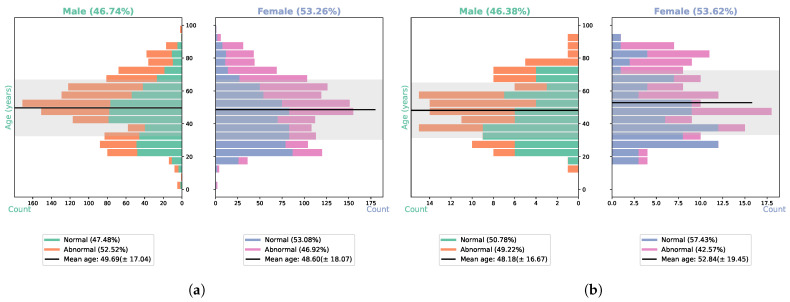
Age distributions in TUAB v2.0.0, (**a**) training subset and (**b**) evaluation subset, with shaded areas representing standard deviations of ages for both male and female participants. This figure highlights the differences in conditions and gender distribution, shown in gray.

The TUH EEG Corpus [48] is the world’s largest and most accessible open-source EEG database. It has enabled numerous studies in the field of EEG analysis, including, but not limited to, seizure detection, seizure type classification, and general abnormality detection in EEG. One subset, TUAB (v2.0.0) [37], was released to enable research into the problem of automating EEG diagnostic evaluation. TUAB comprises 2993 EEG recordings obtained from over 2000 patients at TUH who were diagnosed with conditions such as epilepsy, strokes, depression, and AD [12,13]. The number of channels and sampling rates varied across EEG recordings, with each recorded from at least 21 channels, and most of the data were sampled at 250.0 Hz. Also, the duration for each EEG recording was roughly around 20 min [27,37]. The dataset includes both male and female patients across a wide range of ages (7 days–96 years). TUAB was released with two subsets: training and evaluation. The training set consists of 1488 abnormal and 1529 normal EEG sessions, while the testing set comprises 276 EEG recordings (150 normal/126 abnormal). The recordings underwent verification by human evaluators, resulting in a consensus of 99% for the training subset and a perfect agreement of 100% for the evaluation subset. Table 3 provides the details of the number of recordings and patients, whereas Figure 1 illustrates the age distributions in TUAB v2.0.0 training (a) and evaluation (b) sets, showing differences in normal and abnormal conditions and gender distribution. The training and evaluation sets do not share any record(s) from the same patient, making TUAB an invaluable resource for investigating the potential of techniques for patient-independent automatic detection of abnormality in EEGs.

In addition to TUAB, our study also utilized the TUH EEG Epilepsy Corpus (TUEP) [49] to test the generalizability of our proposed model. This dataset, serving as a separate dataset, consists of EEG recordings acquired from 200 patients (100 epileptic/100 non-epileptic).

### 2.2. Preprocessing

**Figure 2:**
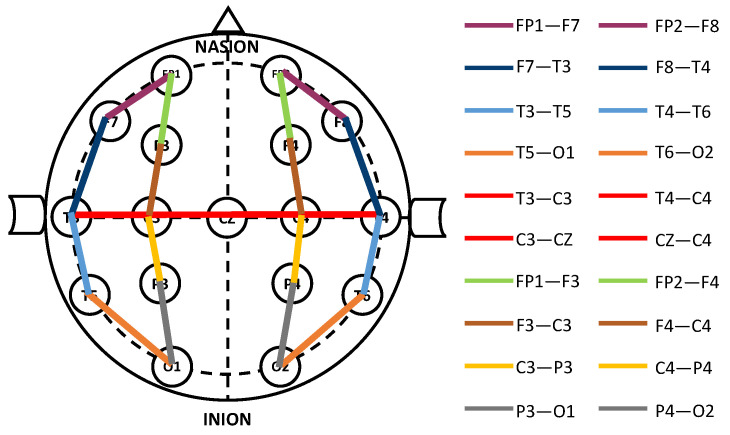
Depiction of EEG channels and their scalp locations following the International 10–20 system, as employed in this research, using TCP montage, adapted from [24].

In this study, we aimed to build a pipeline that eliminates the need for manual feature extraction. However, initial standardization of the data is necessary before feeding it into the deep learning model [13]. For our primary dataset, TUAB, we first applied a fourth-order Butterworth high-pass filter with a 1 Hz cutoff frequency, followed by a notch filter at 60 Hz in order to remove power-line interference and to reduce low-frequency drift and noise [14]. This enhanced the signal-to-noise ratio and improved the overall clarity and precision of our data analysis. We then used the Transverse Central Parietal (TCP) montage technique to accentuate the spike activity within the EEG signal, resulting in 20 EEG channels, as shown in Figure 2 [11,24,37]. Notably, this set of EEG channels was consistently used across all recorded data, ensuring uniformity and comparability of our results. Lastly, all recordings were re-sampled at 250 Hz; this choice was based on the recommendation by previous studies [11,21,37]. Moreover, we augmented the data by using the second 60-second segment to generate additional training samples [11,44]. These were used in reverse sequence to present diverse data representation, a technique known as Time Reverse Augmentation [50]. This method has been proven to improve the performance of deep learning models for classification of EEG data [50,51].

For the secondary dataset, TUEP, we elected to employ the same preprocessing technique as utilized in the study by McDougall et al. [52]. This choice was motivated by our aim to enable direct comparison of our results with the established findings of McDougall et al. The technique can be summarized as follows:

- Selection of EEG data: Only files with significant epileptic activity (IEDs) were used, resulting in 623 files, as shown in Table 3 [52];
- Standardization of channels: Each recording was standardized to include 30 channels, for consistency;
- Utilization of data segments: The first 30 s from each recording were used;
- Application of a bandpass filter: A second-order Butterworth bandpass filter was applied between 0.5 and 49 Hz to remove artifacts.

### 2.3. Proposed Deep Learning Model

Deep neural networks, composed of multiple stacked layers with neurons that mimic human brain activity, have become prevalent in the field of brain electrical activity classification. This includes applications such as seizure detection, classification, and Brain–Computer Interfaces (BCIs). For processing sequential data, RNN-based models such as LSTM and GRU have been widely used [53]. However, these types of models often struggle with limited short-term memory, hindering their ability to learn long-term patterns [53]. A common solution is to shorten input sequences, e.g., through 1D convolutional layers [11,52,53,54]. Alternatively, WaveNet is used, which is another sequential data processing architecture which uses dilated causal convolutions to efficiently extract features and handle long time series or complex patterns [35,55,56].

**Figure 3:**
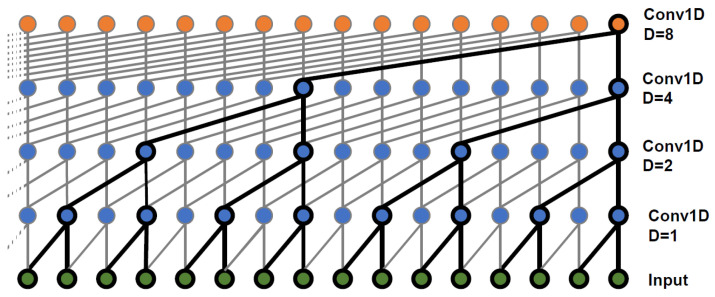
Dilated causal convolutional procedures.

WaveNet was introduced by researchers from DeepMind at Google [35]. The originally proposed WaveNet had the ability to generate raw audio data with high temporal resolution. Experimental results revealed that it significantly outperforms more contemporary methods for Speech-to-Text (STT) and Text-to-Speech (TTS) tasks [57]. WaveNet architecture consists of multiple 1D CNN layers stacked on top of each other with a dilation rate doubled at each layer [53]. The dilation rate, the core element of WaveNet, is the distance between neurons in each layer. Figure 3 shows the process of dilated convolution found in WaveNet architecture. It can be observed that the first convolution layer receives a glimpse of only two time steps of the sequence. In the next layer, double dilation rate, four steps of the sequence are perceived. In the following layer, eight steps occur at a time, and so on. In this way, the network can learn long-term patterns as well as short-term patterns of the input at the higher-level layers and lower-level layers, respectively. Moreover, the design of WaveNet comprises residual blocks with gated activation units and skip connections, which help the network to avoid the vanishing gradient problem, promote convergence, and speed up training.

**Figure 4:**
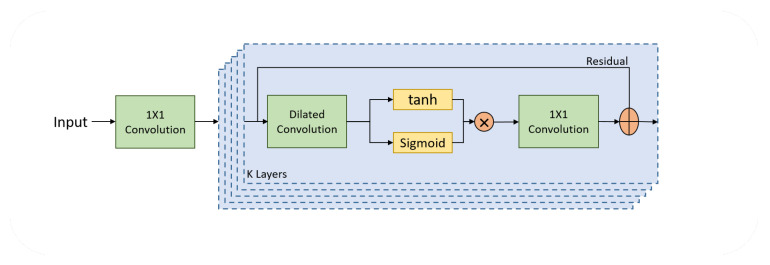
Block diagram of the WaveNet architecture that shows the process of convolution with different dilation rates, as well as residual and gated activation.

Due to the characteristics of WaveNet, we adopted the WaveNet architecture and modified it to use it with EEG data. Figure 4 presents the WaveNet architecture which we utilized in our study. The modified WaveNet architecture was implemented as blocks that employ dilated convolutional layers with gated activation units to process input signals. The architecture first iterates through a range of dilation rates. For each dilation rate, a separate 1D convolutional layer is applied, with tanh and sigmoid activation functions. The gated activation units manage and shape the information flow within the model. They assign weights to the data, based on its importance, to enable the model to better capture the temporal patterns in the input signals. Unlike the original WaveNet, however, we did not use skip connections, in order to provide us with more control over the receptive field size and, thus, the ability to capture various temporal features more effectively. The outputs of the convolutional layers are then accumulated to produce the final output, which contains combined information from all gated activation units.

**Figure 5:**
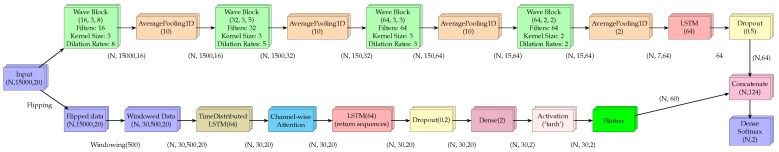
A dual-path neural network architecture, combining modified WaveNet- and LSTM-based paths for effective processing and classification of raw EEG signals. The modified WaveNet sub-model (top path) utilizes a series of Wave Blocks with varying filters, kernel sizes, and dilation rates, followed by AveragePooling1D layers and a final LSTM layer. The LSTM-sub model (bottom path) employs data flipping, windowing, TimeDistributed LSTM, channel-wise attention mechanism, and an LSTM layer with Dropout and dense layer. The outputs from both sub-models are concatenated and passed through a final dense layer with Softmax activation to produce classification results.

Our proposed model is a hybrid architecture that incorporates both modified WaveNet and LSTM. Figure 5 illustrates the overall structure of our proposed model. The first part of the model employs four blocks of the WaveNet architecture to extract features and capture dependencies within input sequences using dilated convolutions. These blocks have different limits of dilation rates, which creates dynamic receptive fields for our model. The first wave block starts with a dilation sequence of [1, 2, 4,..., 128], to enable our model to capture broad temporal patterns [35]. As we move through the subsequent blocks, the dilation rates reduce, focusing more on localized temporal patterns. This approach ensures our model effectively captures both high-level features as well as fine-grained details within the EEG signals. Each wave block is followed by an AveragePooling1D layer to reduce the sequence length. Then, the output is processed by an LSTM layer, leveraging its ability to handle time-dependent information and temporal patterns [30].

To further improve performance, we incorporated a second path, complementing the WaveNet–LSTM combination. This path involves flipping the input data and applying a windowing technique to divide the input into smaller segments. These segments are then independently processed by an LSTM TimeDistributed layer, followed by channel-wise attention, inspired by [58], to enable the model to focus on the most relevant information. The attended signals are then passed through a simple LSTM layer with 64 neurons and a dense layer. The flattened feature map of the dense layer is then merged with the WaveNet–LSTM output for classification. By incorporating this second path, our proposed model addresses potential limitations and significantly enhances its overall ability to process EEG data efficiently, to improve detection of abnormalities.

### 2.4. Model Architecture and Training Details

The proposed model consists of two paths, a WaveNet–LSTM and an attention-based LSTM (see Figure 5). It was built and trained using 50 epochs, with a batch size of 17, and the BalancedBatchGenerator from the imbalanced-learn library to ensure balanced class representation in each batch. Early stopping was used as a method of regularization technique to prevent overfitting. It monitors the validation loss and suspends training when there is no improvement over 10 consecutive epochs [28,58]. The effectiveness and reliability of performance rely on having a balance between a model’s learning capacity as well as its generalizability [53]. Therefore, early stopping is one of the most optimal methods for achieving this balance by preventing over- or under-fitting due to erroneous training epoch specifications [53]. The cross-entropy function served as the cost metric, and we utilized the Adam optimization algorithm. We set the initial learning rate at 0.001 and employed a callback function to adjust the learning rate during training based on the loss function with a minimum value of $10^{-4}$.

Following initial training and evaluation on the training and validation data, we entered an iterative development stage. This involved multiple rounds of training, adjustment, and reevaluation to enhance the model’s performance incrementally before finalizing the model, as described previously.

We trained our proposed model on 70% of the TUAB training set, while the remainder of the set was used as validation data. We report the final results using a hold-out dataset, as shown in Table 3.

### 2.5. Performance Evaluation Metrics

We report the final results of our proposed model on held-out datasets, as shown in Table 3. We computed three widely used metrics in the literature: sensitivity, which measures how well the model identifies abnormal cases; specificity, which reflects the model’s proficiency at detecting normal cases; and accuracy, which provides a measure of the overall performance of our model. These metrics are derived from the confusion matrix, which presents the true positive rate (TP), true negative rate (TN), false positive rate (FP), and false negative rate (FN). Hence, sensitivity, specificity, and accuracy are calculated as follows:

(1) $$\mathrm{Sensitivity}=\frac{TP}{TP+FN}\times 100$$

(2) $$\mathrm{Specificity}=\frac{TN}{TN+FP}\times 100$$

(3) $$\mathrm{Accuracy}=\frac{TP+TN}{TP+TN+FP+FN}\times 100$$

### 2.6. Ablation Studies

To verify the contribution of each component of our proposed model, we conducted a series of ablation studies. We chose the modified WaveNet architecture as our baseline architecture, in which we replaced the LSTM component within the modified WaveNet–LSTM path, with a GlobalAveragePooling layer for simplicity and computational efficiency. The standalone LSTM path was also excluded in this initial setup. Then we progressively added or removed components from this baseline model and observed the changes in performance.

- Ablation Study 1: The model includes only modified WaveNet–LSTM path (Figure 5, top path), without LSTM path;
- Ablation Study 2: The model includes the entire architecture, but without LSTM layer in modified WaveNet–LSTM path, instead replacing it with a GlobalAveragePooling layer. This helps to identify the contribution of the LSTM component in modified WaveNet–LSTM path;
- Ablation Study 3: The model includes only the standalone LSTM path (Figure 5, bottom path), without modified WaveNet–LSTM path. This helps identify the contribution of the standalone LSTM path independently.

## 3. Results and Discussion

**Figure 6:**
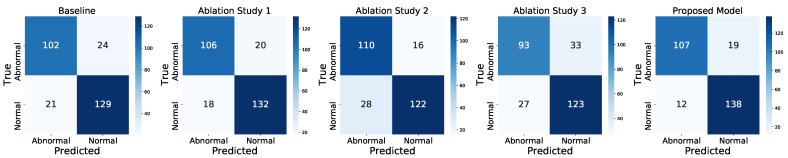
Confusion matrices from the baseline, ablation studies 1–3, and final model, evaluated over the TUHAB evaluation dataset.

Table 4 presents the results of the experiments, while Figure 6 shows the confusion matrices for the baseline model, each ablation study, and the final proposed model, as described in the aforementioned ’Ablation Studies’ sub-section.

It can be observed from the table that the baseline model achieved an accuracy of 83.7%. However, with the addition of an LSTM layer at the end of the baseline model, as in Ablation Study 1, the model’s performance in terms of accuracy improved by 3%, with better rates of true positives and true negatives, as shown in Figure 6. This improvement indicates that the addition of an LSTM layer to the baseline model contributes to a more precise classification result. We attribute this improvement to the ability of LSTM to refine local temporal relations based on the features learned by the dilated convolutions in WaveNet. Specifically, LSTM’s ability to learn long-term dependencies in the data is crucial for capturing patterns in EEG signals, which often involve complex temporal dynamics over time. This integration offers a powerful and flexible solution for processing sequential data to overcome the limitations of each individual architecture.

Having established the importance of the LSTM layer in the baseline model, we then investigated the impact of adding an independent LSTM-based path sub-model in parallel to the baseline model in the second ablation study. This approach has previously demonstrated efficient learning from diverse data representations [5]. Interestingly, the results showed a decrease in accuracy compared to the first ablation study, achieving an accuracy of 84.05%. However, this experiment obtained a high degree of true positive rate, as shown in Figure 6, yielding a sensitivity of 87.3%. Motivated by these promising results of the second ablation study, we proceeded to examine the performance of the entire proposed model, by incorporating all of the previous components. As a result, the final model revealed balanced results between sensitivity and specificity, with the highest classification of abnormality in EEGs. Moreover, we investigated the performance of the standalone LSTM path as a single model. However, the obtained results only reached an accuracy of 78.26%, indicating that this sub-model by itself may not be sufficiently effective for classifying EEG signals. Nevertheless, it contributes cooperatively to the final overall performance of the WaveNet–LSTM sub-model, as illustrated in Figure 6. Overall, our final proposed model, with all of its components, outperforms the other configurations. The best-obtained results are 88.75%, 84.92%, and 92% for accuracy, sensitivity, and specificity, respectively.

These findings confirm the importance of both the modified WaveNet–LSTM and LSTM paths in our model architecture. The final model leverages the strengths of both paths and achieves the highest classification accuracy. This can be credited to the design of the dilated convolutions in WaveNet, which captures multi-scale local patterns in time-series data. Concurrently, the LSTM provides an additional level of feature extraction by refining these local patterns in terms of their temporal relations. Additionally, the standalone LSTM sub-model focuses on extracting long-term dependencies, thereby extending the final learned features by modified WaveNet–LSTM path.

We compared our results with the existing state-of-the-art end-to-end deep learning methods, using the same dataset for the same problem, and presented the results in Table 5. We can observe that our proposed model achieved better results than all other state-of-the-art techniques.

Our proposed method stands out, as it only employed the initial duration, 60 s, of the signals, contrasting with other state-of-the-art research that required significantly longer input signals. Yildirim et al. [43] employed the first 60 s of the signal for classification, and our model improved on their classification results by more than 8%.

Compared to the most recent state-of-the-art models reported in the literature [46,47], our proposed model demonstrates an improvement in accuracy by 2.17%. Most of the other research studies used up to 11 min of EEG data for training [11,12,13,44,45].

Furthermore, our proposed model simplifies the complexity compared to other models, with only 244,992 parameters, as opposed to models with 143 million [17] and 61 million parameters [45].

Additionally, our proposed model achieved a better sensitivity score, indicating a high degree of correct true positive and false negative rates, while maintaining the specificity score of a good degree of true negative and false positive rates, as shown in Figure 6. Correctly identifying positive cases is critical to prevent serious consequences [59,60]. Similarly, it is essential to limit false positives, thus avoiding unnecessary treatments and their associated costs [36,61].

To further demonstrate the robustness and generalizability of our model, we conducted an additional experiment using a separate dataset: TUEP. Notably, this experiment was performed without any adjustments or hyperparameter tuning to the architecture. This means that our proposed model, initially trained solely on the TUAB dataset, was directly applied to the TUEP dataset without any modifications.

We made this decision because our main objective was to test the model’s generalizability, rather than to achieve the highest possible performance on the TUEP dataset. Additionally, this decision was inspired by the previous research in the paradigm of EEG signal analysis [28]. Therefore, we opted to use the same model parameters as those optimized for the TUAB dataset, though hyperparameter tuning may further enhance the model’s performance on TUEP.

This approach allowed us to evaluate how well our model performs on new, unseen data. Impressively, our model achieved an accuracy of 97.45% on the TUEP dataset, compared to an accuracy of 94.92% achieved by [52], which is more than a 3% improvement in terms of accuracy (see Table 6). Furthermore, the obtained results exhibit an almost perfect true positive rate with a very low false negative rate—with only one abnormal case misclassified as normal. These results clearly demonstrate the effectiveness of our proposed model when applied to real-world scenarios.

The use of the BalancedBatchGenerator during training has proven its effectiveness in the case of the unbalanced dataset, as shown in the case of the TUEP dataset. However, it is crucial to explore other techniques, such as up-sampling the minority class and downsampling the majority class; moreover, if the dataset is heterogeneous, other metrics for evaluating the performance could be used, such as F1-score [5,28,62].

Reexamining the results presented previously, we can discuss a couple of points regarding the strength of our proposed model and its components:

- The most important contribution of this study is the use of a combination of WaveNet and LSTM in the classification of normal and abnormal EEG signals, which can be a crucial reason why the proposed model achieved better results than the previous state-of-the-art studies;
- The fusion of LSTM and WaveNet within two distinct paths is another novel aspect of our approach not yet explored in the existing literature. This innovative architectural design reinforces the superiority of our proposed method over state-of-the-art reported approaches;
- Our ablation studies provide valuable insight into the individual contribution of each component in our architecture. The experimental results demonstrate that both LSTM and modified WaveNet–LSTM paths play integral roles in enhancing the performance of the model;
- One of the strongest validations of our proposed model’s effectiveness is its excellent performance on an entirely separate dataset, namely, the TUEP dataset, without any further hyperparameter tuning or adjustments. The model’s high accuracy and low false negative rate on this dataset illustrate its robustness and generalizability on unseen EEG data. This suggests that our model can be effectively applied across different datasets, demonstrating practical value in real-world scenarios.

Our research findings suggest that the proposed model has significant potential for practical EEG classification applications by serving as a preliminary diagnostic tool, especially in areas where there is a scarcity of clinical expertise. However, it is vital to maintain collaborative integration between machine learning technologies and experts to ensure efficacy in real-world scenarios. Importantly, interpreting machine learning models, especially those in decision-making roles within healthcare, is essential to align these applications with established medical principles and practices, which not only validates their performance in accordance with physicians’ clinical knowledge but also promotes their acceptance and trust within the medical community. Additionally, the ethical implications of deploying machine learning models in healthcare are important, particularly regarding accountability, data privacy, and prediction biases. Furthermore, formal verification methods—mathematical approaches to verify the reliability and robustness of software and hardware systems—offer a robust methodology to ensure the correctness of machine learning systems [63,64]. Using such methods can contribute to the safety and trustworthiness of automated systems, in particular, within critical sectors such as healthcare, where the implications of errors can be substantial [63,64]. Thus, future research could focus on integrating these methods into the development process of machine learning applications in order to enhance their reliability in real-world applications.

## 4. Conclusions

A novel deep learning architecture for the automatic detection of abnormal EEG signals is introduced in this paper. Our proposed model incorporates modified WaveNet and LSTM sub-models, providing an effective approach to discerning normal from abnormal EEG signals. The efficacy of our model is substantiated by its performance compared to other state-of-the-art research studies. It achieved an accuracy of 88.76% on the TUAB EEG dataset. Additionally, it demonstrated high sensitivity (84.92%) and specificity (92%) rates, indicating a robust true positive rate and a low false negative rate.

We also performed several ablation studies to verify the significant contribution of each component of our proposed architecture. Furthermore, the model’s generalizability was further evaluated by applying it to the TUEP dataset without any adjustment or hyperparameter tuning. Despite the change in data used, our model maintained high performance, yielding an accuracy of 97.7%, which confirms its robustness when generalized to unseen patients’ EEG data.

In the future, we plan to investigate different input modalities in our proposed model such as ECG and EMG data. This is expected to enhance the effectiveness in detecting abnormalities across a wider range of neurological data.

## Figures and Tables

**Table 1:** Feature-based approaches for abnormality detection in EGG using TUAB dataset.

Studies	Year	Input	Architecture	ACC (%)
Lopez et al. [37] *	2017	band power-based features using Cepstral coefficients	CNN + Multilayer Perception (MLP)	78.8
Alhussien et al. [27] *	2019	FFT band-limited signals	AlexNet + MLP	89.13
Gemein et al. [12]	2020	DWT + CWT + DFT + Statistical features	RG	85.90
Cisotto et al. [40]	2020	Statistical features + spectral power in specific frequency bands	LSTM+attention	79.18
Sharma et al. [41]	2020	Wavelet-based statistical features	SVM	79.34
Albaqami et al. [21]	2021	WPD + Statistical features	CatBoost	87.68
Singh et al. [17]	2021	Spectrogram image based on STFT	VGG-19 + RF	88.04
Bajpai et al. [14]	2021	Spectrogram image based on STFT	SeizNet + SVM	96.56
Mohsenvand et al. [42]	2021	EEG contrastive learning	Simple Contrastive Learning of Visual Representations(SimCLR)	87.45
Wu et al. [16]	2022	Statistical features from DWT coefficients	CatBoost	89.13
Wu et al. [23]	2022	Statistical features from WPD coefficient	Catboost	89.76
Tasci et al. [25]	2023	Multilevel Discrete Wavelet Transform (MDWT) + Statistical features	KNN	87.78
Zhong et al. [38]	2023	Statistical features from WPD coefficients	CatBoost	89.13
Kohad et al. [15]	2022	EMD and EWT based features	Linear SVM	88.48

* Used extra training data not included in TUAB.

**Table 2:** End-to-end machine learning approaches for abnormality detection in EGG using TUAB dataset.

Studies	Year	Input	Architecture	ACC (%)
Schirrmeister et al. [13]	2017	Raw EEG data	Deep CNN	85.42
Roy et al. [44]	2018	Raw EEG data	1D-CNN–RNN	82.27
Amin et al. [45] *	2019	Raw EEG data	AlexNet + SVM	87.32
Roy et al. [11]	2019	Raw EEG data	1D-CNN–GRU ChronoNet	86.57
Yildirim et al. [43]	2020	Raw EEG data	1D-CNN	79.34
Gemein et al. [12]	2021	Raw EEG data	TCN Model	86.16
Khan et al. [46]	2023	Raw EEG data	Hybrid Model (LSTM and CNN)	85.00
Kiessner et al. [47] *	2023	Raw EEG data	Deep CNN [13]	86.59

* Used extra training data not included in TUAB.

**Table 3:** Number of recordings and patients found in TUAB (V2.0.0) and TUEP datasets.

TUAB	Samples		Patients	
	Normal	Abnormal	Normal	Abnormal
Training	1371	1346	1237	893
Evaluation	150	126	184	105
Total	1521	1472	1385	998
**TUEP**	**Samples**		**Patients**	
	**Non-Epileptic**	**Epileptic**	**Non-Epileptic**	**Epileptic**
Training	224	451	80	32
Evaluation	64	172	20	10
Total	288	623	100	42

**Table 4:** Performance of the proposed model and its variants on the TUHAB evaluation dataset.

Study	Accuracy	Sensitivity	Specificity
Baseline	83.69	80.95	86
Ablation Study 1	86.231	84.126	88
Ablation Study 2	84.06	**87.3**	81.33
Ablation Study 3	78.26	73.809	82
Proposed model	**88.76**	84.92	**92**

**Table 5:** Comparison of the proposed method and other state-of-the-art methods on the TUAB dataset.

Study	Accuracy	Sensitivity	Specificity
Yildirim et al. [43]	79.34		
Roy et al. [44]	82.27		
Schirrmeister et al. [13]	85.4	75.1	94.1
Khan et al. [46]	85		
Gemein et al. [12]	86.1	79.7	91.6
Roy et al. [11]	86.57		
Kiessner et al. [47]	86.59	78.17	93.67
Amin et al. [45]	87.32	78.57	94.67
Proposed model	**88.76**	**84.92**	92

**Table 6:** Performance for the proposed model using the TUEP dataset and comparison with previous research using the same dataset.

Study	Accuracy	Sensitivity	Specificity
McDougall et al. [52]	94.92	96.51	90.62
Proposed model	**97.45**	**97.09**	**98.43**

## Data Availability

The data used in this study are obtained from the TUH EEG Corpus. Access to this dataset requires a formal request to the Neural Engineering Data Consortium (NEDC). Detailed instructions on how to submit a request can be found on the official website at: https://www.isip.piconepress.com/projects/tuh_eeg/, accessed on 15 October 2022.
